# An immune-related pseudogene signature to improve prognosis prediction of endometrial carcinoma patients

**DOI:** 10.1186/s12938-021-00902-7

**Published:** 2021-06-30

**Authors:** Shanshan Tang, Yiyi Zhuge

**Affiliations:** Department of Gynecology, Hangzhou Women’s Hospital, No. 369 Kunpeng Road, Shangcheng District, Hangzhou, 310008 Zhejiang China

**Keywords:** Uterine corpus endometrial cancer, Immune, Pseudogene, Survival, Signature

## Abstract

**Background:**

Pseudogenes show multiple functions in various cancer types, and immunotherapy is a promising cancer treatment. Therefore, this study aims to identify immune-related pseudogene signature in endometrial cancer (EC).

**Methods:**

Gene transcriptome data of EC tissues and corresponding clinical information were downloaded from The Cancer Genome Atlas (TCGA) through UCSC Xena browser. Spearman correlation analysis was performed to identify immune-related pseudogenes (IRPs) between the immune genes and pseudogenes. Univariate Cox regression, LASSO, and multivariate were performed to develop a risk score signature to investigate the different overall survival (OS) between high- and low-risk groups. The prognostic significance of the signature was assessed by the Kaplan–Meier curve, time-dependent receiver operating characteristic (ROC) curve. The abundance of 22 immune cell subtypes of EC patients was evaluated using CIBERSORT.

**Results:**

Nine IRPs were used to build a prognostic signature. Survival analysis revealed that patients in the low-risk group presented longer OS than those in the high-risk group as well as in multiple subgroups. The signature risk score was independent of other clinical covariates and was associated with several clinicopathological variables. The prognostic signature reflected infiltration by multiple types of immune cells and revealed the immunotherapy response of patients with anti-programmed death-1 (PD-1) and anti-programmed cell death 1 ligand 1 (PD-L1) therapy. Function enrichment analysis revealed that the nine IRPs were mainly involved in multiple cancer-related pathways.

**Conclusion:**

We identified an immune-related pseudogene signature that was strongly correlated with the prognosis and immune response to EC. The signature might have important implications for improving the clinical survival of EC patients and provide new strategies for cancer treatment.

## Background

Endometrial cancer (EC) was the mainly gynecological malignancy and ranked fourth in women malignancies worldwide behind breast, lung, and colorectal cancers, and there were approximately 61,880 new patients and 12,160 deaths of uterine corpus endometrial cancer in 2019, according to global cancer statistics [1, 2]. Early-stage patients presented a favorable clinical outcome with a 5-year survival rate of 95%. However, patients in advanced stage had a reduced 5-year survival rate of lower than 20% for stage IV [3]. Surgery, adjuvant chemotherapy, radiation, immunotherapy, and hormonal therapy are common models of treatment for patients with EC and have reduced the mortality of patients to a certain extent in recent years. However, despite rapid advances in the treatments of UCEC, the incidence and mortality rates are still increasing since it is a clinically heterogeneous disease characterized by different genetic background and pathogenesis [4]. Thus, to improve the survival rates of UCEC patients, it is imperative to identify mechanistic differences at the molecular level and develop novel predictive biomarkers to predict patient outcomes accurately.

Pseudogenes were initially considered as nonfunctional gene fossils or junk genes, which usually originating from their parent genes but have lost the capacity to encode functional proteins due to the accumulation of gene mutations [5, 6]. In recent years, accumulating evidence has strongly demonstrated that aberrant expression of pseudogenes plays vital roles in many human diseases such as cancer [7, 8]. Previous studies reported that pseudogenes mainly regulate gene expression at the post-transcriptional level and function as positive or negative regulators in tumor initiation or progression via two possible pathways. Numerous pseudogenes can act as competitive endogenous RNAs to competitively bind miRNAs with the protein-coding gene, therefore positively regulating gene expression [9, 10]. In addition, pseudogenes can also play a negative role through completing with their parent genes to destabilize RNA binding proteins, which results in a reduction in parent gene expression [11]. A previous study has revealed that LDHAP5 pseudogene was associated with the poor prognosis of ovarian serous cystadenocarcinoma via its targeting of EGFR [12]. Abnormally activated OCT4 pseudogene 5 (OCT4-pg5) contributed to enhanced cell proliferation by competing with miR-145 in EC via upregulating OCT4 expression [9]. Although a number of studies have identified multiple differentially expressed pseudogenes in various cancers, the generally predictive roles of immune-related pseudogenes in EC remain unclear. Since no studies have systematically evaluated immune-related pseudogenes in EC, discovering a number of promising prognostic biomarkers and exploring the underlying molecular mechanisms are eagerly needed. Therefore, for the first time, we identified an immune-related pseudogene signature in EC.

In this study, we screened the immune-related pseudogenes found in EC and explored the relationship between the screened immune-related pseudogenes and the prognosis of EC. A signature of immune-related pseudogenes in EC was developed using multivariate Cox regression analysis. Also, we investigated the prognostic value of this signature in various clinical groups and the potential role of the immune-related pseudogenes signature in immune checkpoint inhibitors immunotherapy, with the aim of using it as a promising immune therapeutic target.

## Discussion

With the rapid development of next-generation sequencing, growing transcriptomic data from the public database such as TCGA could be easily acquired. Through mining the big data from public databases, increasing studies have demonstrated pseudogenes, lncRNAs, miRNAs, and circRNAs play important roles in various cellular functions, including proliferation, cell differentiation, DNA stability, and tumorigenesis [9, 13, 14]. As a special group of lncRNAs, pseudogenes are remnants of their parental genes that lost the ability to encode proteins [15]. Accumulating evidence demonstrates crucial roles for pseudogenes in multiple cellular processes and various cancers [16].

In this study, we systematically gathered data from the TCGA portal and extracted immune-related pseudogenes via Spearman correlation analysis. Subsequently, we identified nine prognostic immune-related pseudogenes through univariate, LASSO, and multivariate Cox regression analyses and used them to develop a signature risk score, which was capable of classifying patients into the high-risk and low-risk groups with significantly different OS. Patients in the high-risk group presented shorter OS than patients in the low-risk group. As a heterogeneous disease with multiple clinicopathological features and risk factors, stratification analyses should be performed to confirm whether the signature was robust. The results illustrated that signature could well distinguish patients with all subgroups. In clinical practice, if we obtained the expression of the nine immune-related pseudogenes, the risk scores can be computed based on the coefficients. As a result, whether the patients are classified as low or high risk could be determined; thus the prognosis of patients could be predicted. Compared with four previous signatures, the AUC for 5-year survival and principal components analysis (PCA) illustrated that the present signature had powerful predictive ability. The risk signature was strongly correlated with age, grade, stage, and neoplasm status. The risk score remained an independent prognostic factor by combination with other clinicopathological characteristics via univariate and multivariate Cox regression analyses. Besides, KEGG enrichment analysis illustrated that the immune-related pseudogene signature might be associated with multiple well-known cancer-related pathways, including the choline metabolism in cancer, endometrial cancer, cell cycle, prostate cancer pathways. Previous studies demonstrated obvious differences in choline and lipid metabolism and protein expression patterns between breast and prostate cancer cells in culture and in cancers derived from these cells [17, 18]. A recent study formed a five-gene signature related to the cell cycle that can predict OS for gastric cancer, which was useful for elaborating cell cycle mechanisms and for identifying patients with gastric cancer with poor OS [19]. As for EC, Liu et al. identified five cell cycle-related genes that were notably dysregulated between EC and normal tissues, and the signature based on the 5-cell cycle genes could predict EC prognosis exactly and independently [20]. These further indicated the important role of immune-related pseudogenes in EC.

Tumor microenvironment immune cells infiltration has been regarded as vital information for predicting the outcome and immunotherapy response in various malignancies according to the clinical trials with immune checkpoint inhibitors [21, 22]. The complex interaction between tumor cells and tumor microenvironment not only plays a critical role during the development of tumor, but also has significant effects on the efficacy of immunotherapy and the overall survival rate of patients [23]. Therefore, by applying the newly developed algorithm “CIBERSORT”, the immune cell infiltration levels of patients between high-risk and low-risk groups were assessed. We uncovered a significant difference between two risk groups in terms of CD8^+^ T cell, follicular helper T cells, regulatory T cell, gamma delta T cells, resting dendritic cells, and activated dendritic cells. We further analyzed the relationship between the signature risk score and immune cell infiltration abundance. The results demonstrated a significant positive/negative correlation between the risk score and multiple immune cells, such as activated myeloid dendritic cell, B cell naïve, activated mast cell, gamma delta T cell, regulatory T cell, and CD8^+^ T cell. This is consistent with the viewpoint that T cell and B cell responses play critical roles in diagnosis, prognosis, and survival of cancer patients [24, 25]. Immune checkpoint inhibitors have opened a new era of tumor immunotherapy. Immune regulation against immune checkpoints can result in cancer cell death by providing immune response signals to T cells [26]. The expression of immune checkpoint genes (PD-1, PD-L1, and CTLA-4) has been commonly used as predictive biomarkers for immune checkpoint inhibitors response [27, 28]. PD-L1 and PD-1 are expressed on human B cells and validated to act immunosuppressive roles in cancer progression [29]. In our study, CTLA4 and PD1 were higher expressed in a low-risk group than those in the high-risk group. Furthermore, the risk score was significantly negatively correlated with CTLA4 and PD1 expression. These findings, taken together with survival analysis, suggested that CTLA4 and PD1 have closely participated in immunosuppression, and their high expression is correlated with poor prognosis. Furthermore, we also found that patients with higher IPSs in the low-risk group tended to be candidates for immune checkpoint inhibitor. This demonstrated that the immune-related pseudogene signature is associated with an immunosuppressive phenotype and could be a potential predictive marker for immune checkpoint inhibitor response.

The nine immune-related pseudogenes were novel biomarkers of EC and had vital prognostic significance. The immune-related pseudogenes signature may serve as a new potential biomarker for immunotherapy and contribute to new therapeutic strategies. Furthermore, this study has opened many avenues for future research to pursue this topic. However, there are several limitations that should be noted. First, the number of the clinicopathological parameters released in publicly available datasets is limited and not comprehensive. The signature should be adequately validated in more independent cohorts with larger amounts of patients. Besides, the main research analysis approach we used is based on bioinformatics technology and further cellular experiments and animal studies (in vitro and in vivo) should be conducted to explore the predictive accuracy of the signature and to identify potential immune-related mechanisms in EC. These conclusions need to be further validated for clinical application with additional experimental data, such as flow cytometry or immunohistochemistry. Despite these limitations, to our knowledge, this is the first study to focus on the immune-related pseudogenes signature of EC.

## Conclusion

In conclusion, we proposed an immune-related pseudogenes signature, which can be used as an independent prognostic biomarker in stratifying risk subgroups in terms of OS for patients with EC. The nine-pseudogene signature had a superior performance for risk stratification compared to two existing signatures. The immune-related pseudogenes signature can assess survival and immune checkpoint inhibitor response of patients with anti-PD-1 and anti-CTLA4 therapy, potentially enabling more personalized and precise tumor immunotherapy in the future.

## Methods

### Acquisition of EC expression and clinical data

The three-level RNA-Seq expression profiles data and the corresponding clinical data were retrieved from the public TCGA portal (https://cancergenome.nih.gov/). The probe IDs were converted to the corresponding gene symbols based on their annotation files. When several probes matched to an identical gene symbol, we averaged them for further analysis. Briefly, genes were identified as protein-coding genes or pseudogenes based on their Ensembl IDs in the Ensembl database (https://www.ensembl.org/). The data of genes or pseudogenes expression matrix were obtained. Patients without complete overall survival (OS) time were excluded from this study. Finally, we enrolled expression and clinical data of 541 EC patients.

### Immune-associated pseudogenes acquisition

We acquired 1793 unique immune-related genes from the ImmPort database (https://immport.niaid.nih.gov). Subsequent, the immune-related genes matrix was extracted from the mRNA expression profile. A total of 13,602 pseudogenes were downloaded from the HUGO Gene Nomenclature Committee (https://www.genenames.org/). The relationship was calculated based on the expression value between pseudogenes and immune-related genes. Next, Spearman correlation analysis was conducted between immune-related genes matrix and pseudogenes expression levels in samples to identify immune-related pseudogenes according to the correlation coefficients and *P* values (|correlation coefficient|> 0.4, *P* < 0.001). Then, the expression matrix of immune-related pseudogenes in the TCGA database was extracted.

### Construction of a risk score based on immune-related pseudogenes

To confirm the survival of immune-related pseudogenes, a risk score was designed to construct a unitive signature for EC. First, the univariate Cox regression analysis was performed for all possible immune-related pseudogenes to identify prognostic pseudogenes with significant prognostic value. Pseudogenes with *P* < 0.01 were screened for subsequent analysis. We further narrowed the gene range after the univariate analysis by performing least absolute shrinkage and selection operator (LASSO)-penalized Cox regression analysis with 10-times cross-validations using the glmnet package in R. An optimal risk signature was constructed by performing the stepwise regression multivariate Cox analysis with Akaike information criteria (AIC) algorithm among the prognostic immune-related pseudogenes using the “glmnet” and “survival” packages. Final immune-related pseudogenes and their corresponding coefficients with the smallest AIC value were identified to form the prognostic signature in EC. The signature risk score of each sample was calculated using the following algorithm: Risk score = βpseudogene_1_ * exprpseudogene_1_ + βpseudogene_2_ * exprpseudogene_2_ + ··· + βpseudogene_n_ * exprpseudogene_n_. The risk score was computed by a linear combination of the expression level of lncRNAs weighted by the regression coefficient (β). Exprgene refers to the expression of immune-related pseudogenes in the sample, and β indicates the regression coefficient derived from multivariate Cox analysis. The β was calculated by log-transformed hazard ratio (HR) derived from multivariate Cox regression analysis. Based on the median risk score of the signature, all patients were separated into high- or low-risk groups. Principal component analysis (PCA) was carried out to profile expression patterns of grouped samples. Kaplan–Meier curve analysis, time-dependent ROC analysis, and patients’ survival distribution were performed.

### Independence of the signature risk score and clinical relationship with other clinicopathological features

We compared the relationship between a single pseudogene's expression level in the signature and clinicopathological variables to explore the impact of pseudogene on EC deeply. Next, to explore whether the signature risk score and clinicopathological variables were independent prognostic factors, we conducted univariate and multivariate Cox regression analyses for each factor.

### Immune cell subtypes and its correlation with signature risk score

To further explore the differences between two risk groups in the abundance of infiltrating immune cells from gene expression profiles in EC, we used the CIBERSORT algorithm (https://cibersortx.stanford.edu/) coupled with a set of gene expression matrix features of 22 leukocyte subtypes (LM22) that distinguished 22 immune cell subpopulations from CIBERSORT to calculate immune cell infiltrations [9, 30]. We used the mRNA expression matrix data as the input files to evaluate the immune infractions of each sample through the CIBERSORT algorithm. The number of permutations was set to 1,000, and samples with a CIBERSORT output *P* < 0.05 were used for further analysis. For each sample, the sum of all estimated subpopulations is equal to 1.

### Analysis of immune‑checkpoint inhibitors response through immunophenoscore analysis

Immunomodulators or checkpoints, major histocompatibility complex molecules, effector cells, and immunosuppressive cells are four leading categories that determine the immunogenicity of cancer, which could be evaluated as immunophenoscore (IPS). IPS is calculated without bias using machine learning algorithms with z-scores ranged from 0 to 10 based on the gene expression in representative cell types, where higher z-scores are positively correlated to enhanced immunogenicity. IPS is computed using a scale with a range of 0–10 based on representative cell type gene expression z-scores, where higher scores are associated with increased immunogenicity. The IPS of patients with EC was retrieved from The Cancer Immunome Atlas (TCIA) (https://tcia.at/home) [31].

### Functional enrichment analysis

We examined the co-expressed protein-coding genes between identified pseudogenes and genes expression matrix by calculating the Spearman correlation coefficient through expression profiles in 541 patients with EC (|correlation coefficient|> 0.4, *P* < 0.001). KEGG (Kyoto Encyclopedia of Genes and Genomes) enrichment analysis of immune-related pseudogenes was performed to explore potential biological pathways that immune-related pseudogenes may be participated in.

### Statistical analysis

Wilcoxon rank-sum test was performed to compare the differential abundances of immune infiltrates between low- and high-risk groups, which were presented with *P*-value by “vioplot” package. The Student’s t-test was used to test the expression changes of immune‑checkpoint genes between two risk groups. We used the Chi-squared test to investigate the survival differences between the high-risk and low-risk groups. Kaplan–Meier (KM) survival curves and log-rank test were used to assess differences in survival between two risk groups using the “survminer” package. A time-dependent ROC analysis was used to compare the predictive accuracy. Univariate and multivariate Cox regression analyses were performed based on the “survival” package to identify EC's independent prognostic factors. A *P* value < 0.05 was thought to be significant.

## Results

### Identification of immune‑related pseudogenes and establishment of a signature

**Fig. 1:**
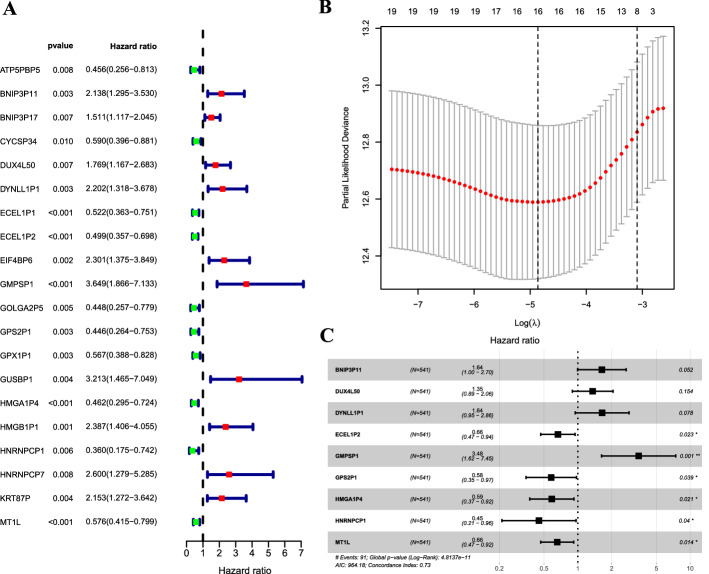
Identification of a nine-pseudogene signature significantly associated with OS of patients with EC. **A** Univariate Cox regression analysis identifying prognostic pseudogenes with HR with 95% CI and P values; **B** a partial likelihood deviance for the LASSO coefficient profiles plot was generated against the log (lambda) sequence; **C** forest plots illustrating the associations of identified nine pseudogenes with OS

By conducting the correlation analysis based on |correlation coefficient|> 0.4 and *P* < 0.001 between immune-related gene and pseudogenes expression matrixes, a total of 312 immune‑related pseudogenes were screened. Univariate Cox regression was used to analyze the prognostic pseudogenes of EC. A total of 20 pseudogenes were significantly relevant to the OS of EC (Fig. 1A). After minimizing overfitting by the LASSO regression algorithm, 16 pseudogenes were entered into the candidate pool for further analysis (Fig. 1B). To determine the optimal prognostic pseudogenes, we adopted stepwise multivariate Cox proportional hazards regression to establish the risk score. Nine pseudogenes entered the final model (Fig. 1C, Table 1). The risk score of the signature for each sample was calculated as the followed equation: risk score = 0.493652865* expression of BNIP3P11 + 0.3035749* expression of DUX4L50 + 0.497475173* expression of DYNLL1P1 − 0.408662104* expression of ECEL1P2 + 1.246821769* expression of GMPSP1 − 0.545480258* expression of GPS2P1 − 0.533330167* expression of HMGA1P4 − 0.797024483* expression of HNRNPCP1 −  0.422476955* expression of MT1L. The risk score of each sample was computed according to the equation. Based on the median risk score of risk score, all patients were divided into low-risk and high-risk groups. The clinicopathological variables in the two risk groups are displayed in Table 2.

**Table 1:** Demographics and clinicopathological characteristics in high-risk and low-risk groups

Variables	Subgroup	High-risk group (*n* = 270)	Low-risk group (*n* = 271)
Age			
	< 65 years	116	171
	> = 65 years	153	99
	Unknown	1	1
Stage			
	Stage I	141	197
	Stage II	28	23
	Stage III	81	42
	Stage IV	20	9
Grade			
	Grade I	18	80
	Grade II	39	81
	Grade III	203	109
	Unknown	10	1
Neoplasm			
	With neoplasm	60	18
	Without neoplasm	188	238
	Unknown	22	15
Survival			
	Alive	196	254
	Dead	74	17

**Table 2:** The nine pseudogenes identified from multivariate Cox regression analyses

Gene symbol	Coefficient	HR	95% CI	*P*-value
BNIP3P11	0.493652865	1.6383	0.9956–2.6959	0.0521
DUX4L50	0.3035749	1.3547	0.8929–2.0554	0.1535
DYNLL1P1	0.497475173	1.6446	0.9466–2.8573	0.0776
ECEL1P2	− 0.408662104	0.6645	0.4675–0.9446	0.0227
GMPSP1	1.246821769	3.4793	1.6245–7.4516	0.0013
GPS2P1	− 0.545480258	0.5796	0.3455–0.9721	0.0387
HMGA1P4	− 0.533330167	0.5866	0.3731–0.9224	0.0209
HNRNPCP1	− 0.797024483	0.4507	0.2105–0.9648	0.0402
MT1L	− 0.422476955	0.6554	0.4676–0.9186	0.0142

HR: hazard ratio; CI: confidence interval

### The performance of pseudogenes signature

**Fig. 2:**
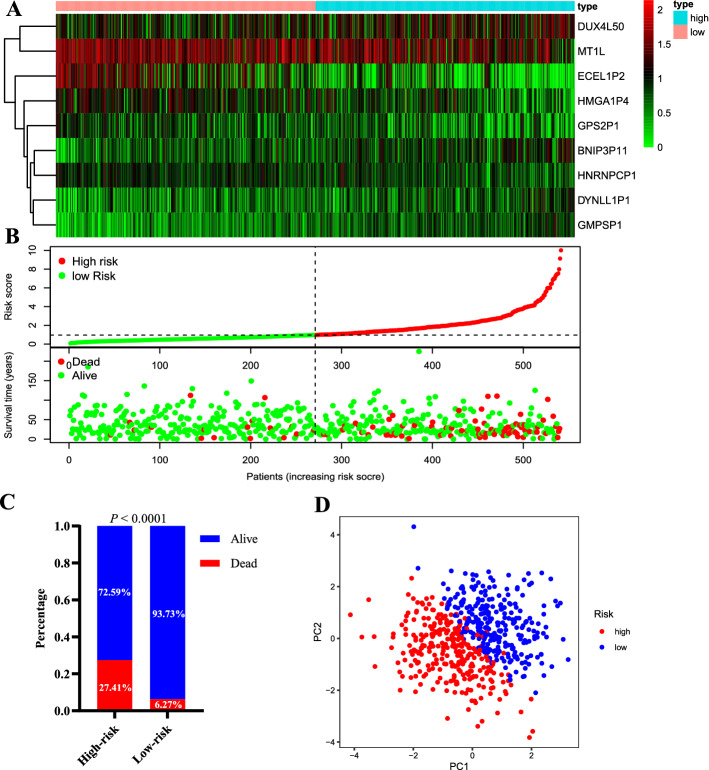
Prognostic risk score signature analysis of seven prognostic genes in HCC patients and the expression level in the low- and high-risk groups. **A** Heatmap illustrated the expression profiles distribution of the signature in the low-risk group and high-risk groups; **B** the distributions of the risk score and patients’ survival status in the low- and high-risk groups; **C** comparison of risk of mortality between the low- and high-risk groups; **D** principal components analysis between low- and high-risk groups based on the nine-pseudogene signature

**Fig. 3:**
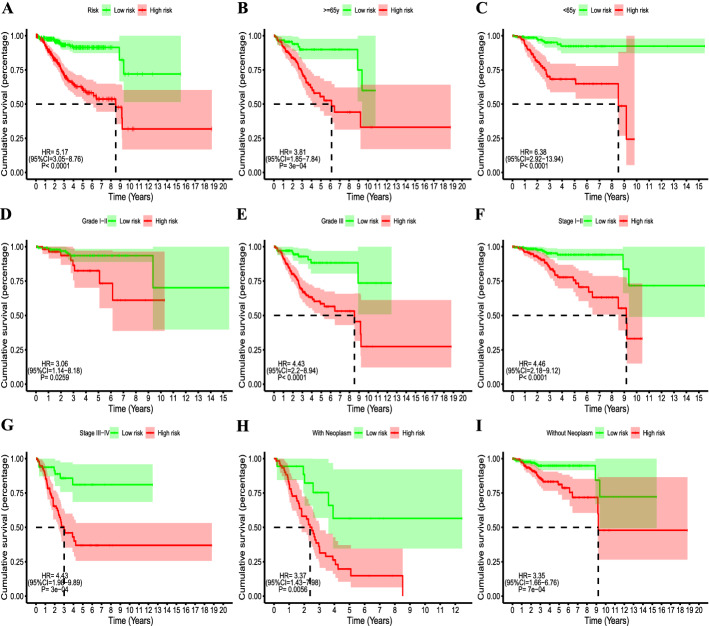
Kaplan–Meier survival analysis curves between the low- and high-risk groups. The survival analysis of patients’ OS in the whole cohort (**A**), age ≥ 65 (**B**), age < 65 (**C**), grade I–II (**D**), grade III (**E**), stage I–II (**F**), stage III–IV (**G**), with neoplasm (**H**), and without neoplasm (**I**)

The mRNA expression heatmap, signature risk score and survival status distribution of each patient are illustrated in Fig. 2A, B. With the risk score increased, more patients died. As revealed in Fig. 2C, patients in the high-risk group have a greater risk of mortality than those in the low-risk group (*P* < 0.0001). Additionally, PCA was used to explore the different distribution patterns between low- and high-risk groups on the basis of the immune-related pseudogenes. PCA analysis of the samples indicated that the clustering of the samples demonstrating a significant distinction between high- and low-risk groups (Fig. 2D). Therefore, immune-related pseudogenes were used to divide the EC patients into two categories, revealing that the immune status of the EC patients in the high-risk group was distinguishable from that in the low-risk group. Patients in the high-risk group illustrated shorter overall survival (OS) than the low-risk group (HR = 5.17, 95% CI 3.05–8.76; *P* < 0.0001; Fig. 3A). These findings were further validated in multiple subgroups. As shown in Fig. 3B-I, the Kaplan–Meier curves demonstrated that significantly worse OS was observed in high-risk patients stratified by age (< 65 years or ≥ 65 years), grade (male or female), stage (stage I–II or stage III–IV), and neoplasm status (with or without), indicating that the signature was a stable prognostic biomarker for patients with EC.

### Correlation between the risk score and clinicopathological characteristics

**Fig. 4:**
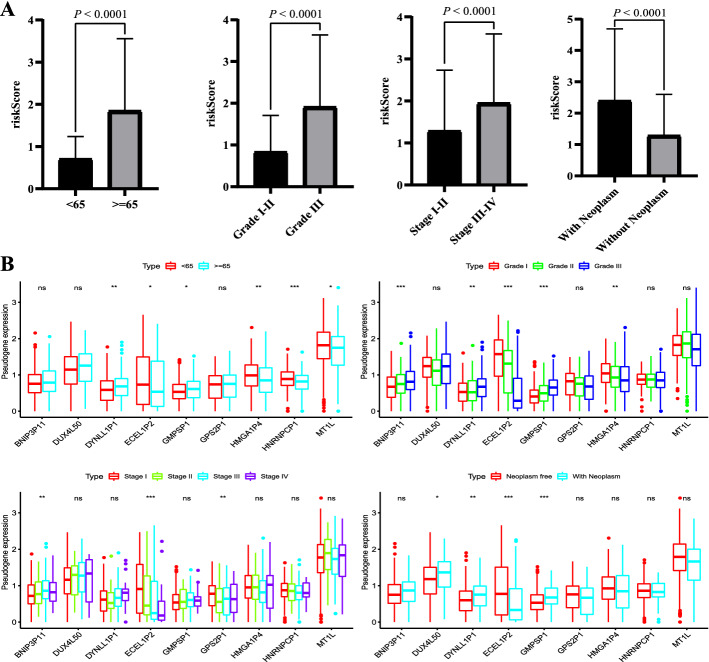
The relationship between the risk score and different clinicopathological features as well as correlation between the expression level of a single pseudogene in the signature and clinical features. **A** represents the relationship between the risk score and age, grade, stage, and neoplasm, respectively; **B** represents the correlation between the expression level a single pseudogene in the signature and age, grade, stage, T stage, and neoplasm, respectively. NS = not significant. *P < 0.05, **P < 0.01, and ***P < 0.001

We analyzed the association between the immune-related pseudogene signature and clinicopathological factors. Significant differences were observed in age (< 65 years or ≥ 65 years), grade (male or female), stage (stage I–II or stage III–IV), and neoplasm status (with or without) (all *P* < 0.0001; Fig. 4A). Next, we compared the correlation between the expression level of a single pseudogene in the signature and clinicopathological characteristics to investigate the impact of pseudogenes on EC. There was a significant difference in the distribution of expression levels of multiple pseudogenes among age (< 65 years or ≥ 65 years), grade (male or female), stage (stage I–II or stage III–IV), and neoplasm status (with or without) (Fig. 4B), especially for the pseudogene ECEL1P2.

### Performance comparison of the pseudogene signature with four existing signatures in survival prediction

**Fig. 5:**
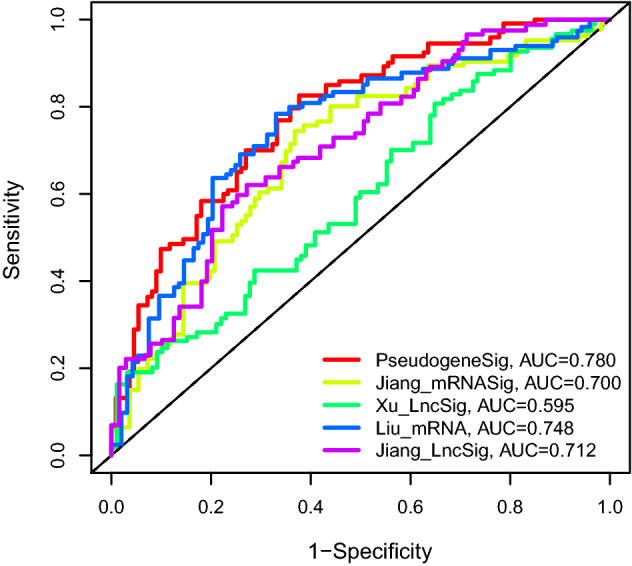
The receiver operating characteristic (ROC) analysis at 5-year of OS in EC for our nine-pseudogene signature and four previous models using the same TCGA cohort

We compared the prediction performance of the novel immune-related pseudogene signature with four recently published signatures: nine-lncRNA signature (LINC02387, FUT8-AS1, UBXN10-AS1, LINC00473, AL353194.1, FAM222A-AS1, AP002761.3, AL731566.2, and AP001021.2) derived from Xu’s research [9], nine-mRNA signature (CYP4F3, LYPLA2, CEL, PHGDH, GPAT3, HNMT, UCK2, CKM, and ACACB) derived from Jiang’s study [32], nine-mRNA signature (TP53, RAE1, RFC2, TAF10, DDB2, UMPS, TAF12, ERCC2, SEC61A1) derived from Liu’s research [33], and five-lncRNA signature (AL121906.2, BOLA3-AS1, LINC01833, AC016405.3, and RAB11B-AS1) from Jiang’s study [34] using the same TCGA EC patient cohort. As illustrated in Fig. 5, the AUC at 5-year of OS for our pseudogene signature is 0.780, which is significantly higher than that of Jiang’s mRNA signature (AUC = 0.7), Xu’s lncRNA signature (AUC = 0.595), Liu’s mRNA signature (AUC = 0.748), and Jiang’s lncRNA signature (AUC = 0.712). Additionally, the number of pseudogenes included in our signature is smaller than that included in the two existing signatures. These results revealed the better prognostic performance of the immune-related pseudogene signature in predicting OS than previously published signatures.

### Independence of the immune-related pseudogenes signature from clinicopathological characteristics

**Fig. 6:**
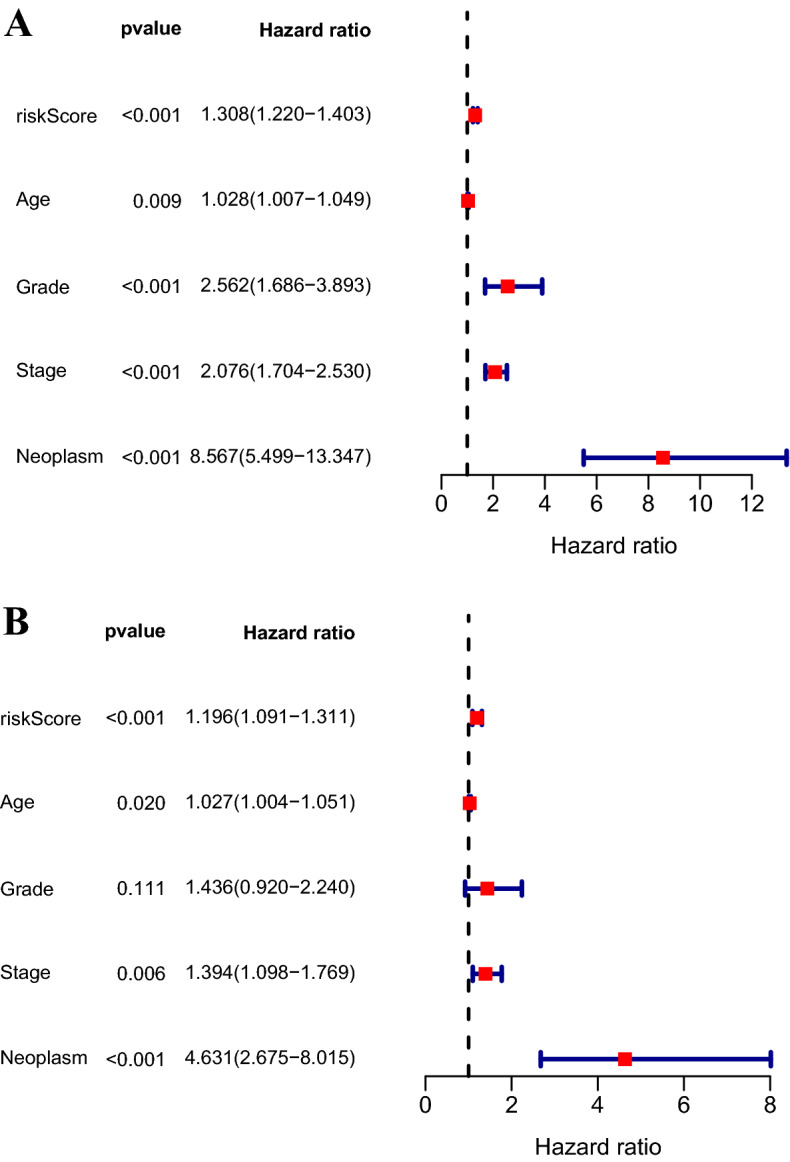
The Cox regression analysis for identifying the independent prognostic value of the risk score. Univariate (**A**) and multivariate (**B**) Cox regression analysis of overall survival for patients with EC

To further explore whether the pseudogene signature could predict OS independently of other clinicopathological factors, univariate and multivariate Cox regression analyses were conducted. We first conducted a univariate analysis of pseudogene signature and other potentially clinicopathological characteristics, and the results illustrated that all the factors were significantly associated with OS (all *P* < 0.05; Fig. 6A). Then, we performed multivariate Cox regression analysis of them, and the results revealed that the pseudogene signature risk score (HR = 1.196, 95% CI 1.091–1.311, *P* < 0.001), stage (HR = 1.394, 95% CI 1.098–1.769, *P* = 0.006), age (HR = 1.027, 95% CI 1.004–1.051, *P* = 0.02), and neoplasm (HR = 4.631, 95% CI 2.675–8.015, *P* < 0.001) could serve as independent prognostic factors for EC patients (Fig. 6B).

### Estimation of immune cell type fractions

**Fig. 7:**
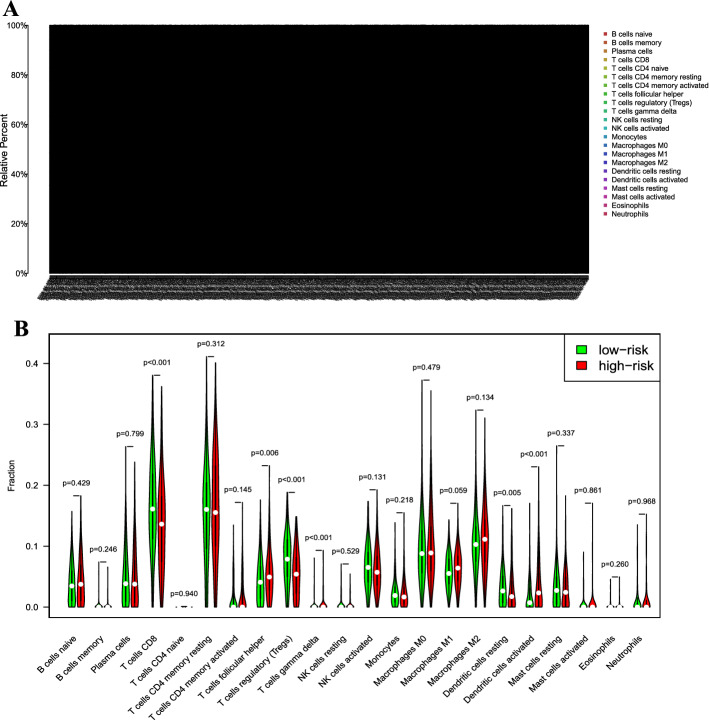
Comparisons of 22 types infiltrating immune cells between low- and high-risk groups. **A** The specific 22 immune fractions indicated by various colors in each patient. **B** Wilcoxon rank-sum test revealed the infiltration levels of various infiltrating immune cells between low- and high-risk groups

**Fig. 8:**
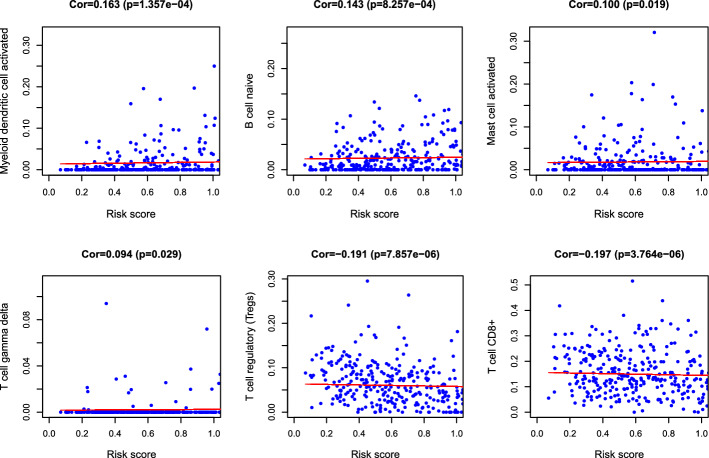
Relationships between the immune-related pseudogene prognostic model and infiltration abundances of six types of immune cells

Previous studies have demonstrated that infiltrating immune cells are strongly related to the prognosis and treatment of malignant cancers [35–37]. Using CIBERSORT, we compared the differences in the immune infiltration of 22 immune cell types between low- and high-risk groups. First, a bar plot was used to illustrate the fractions of 22 immune cells in each sample (Fig. 7A). Next, we analyzed the tumor-infiltrating cells between high- and low-risk subgroups. The results exhibited that CD8^+^ T cell, follicular helper T cells, regulatory T cell, gamma delta T cells, resting dendritic cells, and activated dendritic cells were was significantly different between the two risk groups (all *P* < 0.05; Fig. 7B), which might help to predict immune response and survival. To explore whether immune-related pseudogene signature effectively reflected the tumor immune microenvironment status, the associations between the risk score and infiltration abundances of 22 types of immune cells were analyzed (Fig. 8). The risk score was significantly correlated with activated myeloid dendritic cell (correlation = 0.163, *P* = 1.357e−04), B cell naïve (correlation = 0.143, *P* = 8.257e−04), activated mast cell (correlation = 0.10, *P* = 0.019), gamma delta T cell (correlation = 0.094, *P* = 0.029), regulatory T cell (correlation =  − 0.191, *P* = 7.857e−06), CD8^+^ T cell (correlation =  − 0.197, *P* = 3.764e − 06).

### The immune-related pseudogene signature predicts responses of immunotherapy

**Fig. 9:**
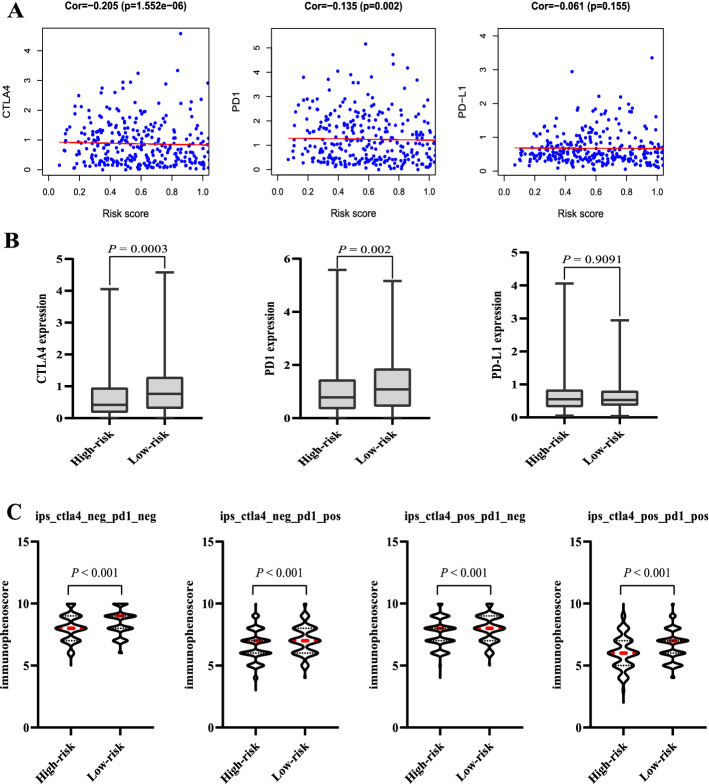
Pearson’s correlation analysis, the expression changes, and IPS analysis between three immune checkpoint genes and the risk score. **A** Pearson’s correlation analysis between the CTLA4, PD1, and PD-L1 expression and the risk score; **B** boxplots of three immune checkpoint genes expression in the high- and low-risk groups stratified by the risk score; **C** IPS comparison between high-risk and low-risk groups in patients with EC in four types. PD-1_pos or CTLA4_pos indicated anti- PD-1 or anti-CTLA4 therapy, respectively

The association between the risk score and expression levels of three immune checkpoint genes was explored. The risk score was significantly negatively correlated with CTLA4 (Spearman correlation coefficient = − 0.205, *P* = 1.552e−06) and PD1 (Spearman correlation coefficient = − 0.135, *P* = 0.002; Fig. 9A). The expression changes of three immune checkpoint genes were compared between the high- and low-risk groups, and the results illustrated that patients with low-risk showed significantly higher expression levels compared with those in the high-risk group (*P* = 0.0003 for CTLA4, and *P* = 0.002 for PD1; Fig. 9B). These findings were consistent with previous views that the immune checkpoint genes closely participated in immunosuppression, and their high expression is correlated with poor prognosis [38]. The relationship between IPS and immune-related pseudogene signature was further explored. The IPS, IPS-CTLA4, IPS-PD1, and IPS-PD1-CTLA4 scores were computed to investigate the signature and predict potential effects on immune checkpoint inhibitor for patients with EC. In the low-risk group, the scores were notably higher than high-risk group (IPS, *P* < 0.001; IPS-CTLA4, *P* < 0.001; IPS-PD1, *P* < 0.001; and IPS-PD1-CTLA4, *P* < 0.001; Fig. 9C). These results revealed that patients presenting higher IPSs in the low-risk group promised to be candidates for immune checkpoint inhibitor.

### Functional annotation of immune‑related pseudogenes

**Fig. 10:**
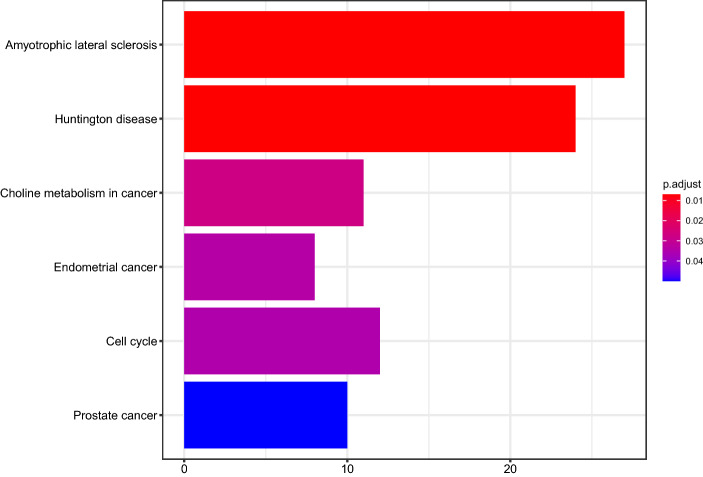
Kyoto encyclopedia of genes and genomes pathway analysis of co-expressed protein-coding genes related to the nine immune-related pseudogenes

We computed the expression correlation between identified pseudogenes and potential protein-coding genes by calculating the Spearman correlation coefficient through expression profiles in 541 patients with EC. Co-expression analysis results identified 857 genes that correlated with nine pseudogenes. KEGG pathway enrichment illustrated that immune‑related pseudogenes were mainly involved in choline metabolism in cancer, endometrial cancer, cell cycle, prostate cancer pathways (Fig. 10).

## Data Availability

The raw data of this study are derived from ImmPort database (https://immport.niaid.nih.gov) and the TCGA database (https://portal.gdc.cancer.gov/), which are publicly available databases.
